# The preventive and therapeutic role of *Lactobacillus* spp. in in vitro model of inflammation via affecting autophagy signaling pathway

**DOI:** 10.1002/iid3.1336

**Published:** 2024-08-27

**Authors:** Fatemeh Haririzadeh Jouriani, Mahnaz Torfeh, Mahdi Torkamaneh, Amin Sepehr, Mahdi Rohani, Shadi Aghamohammad

**Affiliations:** ^1^ Department of Bacteriology Pasteur Institute of Iran Tehran Iran

**Keywords:** autophagy, gut microbiome, inflammation, *Lactobacillus* spp.

## Abstract

**Background:**

Intestinal inflammation has various causes and leads to some inflammatory diseases, of which autophagy pathway dysfunction could be considered as one of them. Probiotics could have a positive effect on reducing inflammation by activating the autophagy pathway. To evaluate the precise effects of probiotics as preventive and therapeutic agents to control the symptoms of inflammatory diseases, we aimed to investigate the efficacy of *Lactobacillus* spp. in regulating the autophagy signaling pathway.

**Methods:**

A quantitative real‐time polymerase chain reaction assay was used to analyze the expression of autophagy genes involved in the formation of phagophores, autophagosomes, and autolysosomes after exposing the HT‐29 cell line to sonicated pathogens and adding *Lactobacillus* spp. before, after, and simultaneously with inflammation. A cytokine assay was also accomplished to evaluate the interleukin (IL)‐6 and IL‐1β level following the probiotic treatment.

**Results:**

*Lactobacillus* spp*.* generally increased autophagy gene expression and consumption of *Lactobacillus* spp. before, simultaneously, and after inflammation, ultimately leading to activate autophagy pathways. The proinflammatory cytokines including IL‐6 and IL‐1β decreased after probiotic treatment.

**Conclusions:**

Our native probiotic *Lactobacillus* spp. showed beneficial effects on HT‐29 cells by increasing autophagy gene expression and decreasing the proinflammatory cytokines production in all treatments. Therefore, this novel probiotic cocktail *Lactobacillus* spp. can prevent and treat inflammation‐related diseases.

## Graphical abstract

*Lactobacillus* spp. showed beneficial effects on HT‐29 cells by increasing autophagy gene expression in all treatments, therefore, *Lactobacillus* spp. can prevent and treat inflammation‐related diseases.

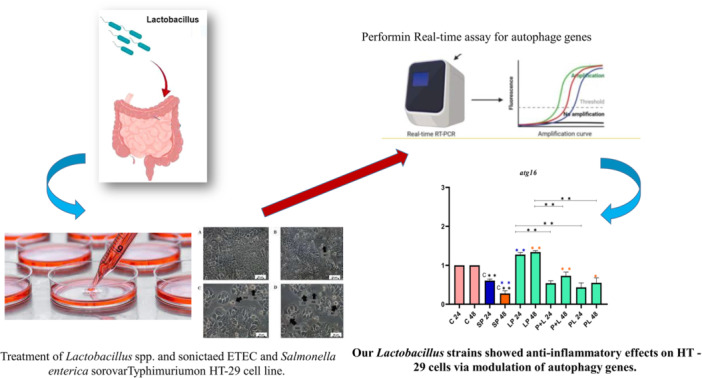

## 1 INTRODUCTION

Probiotics are living microorganisms that their protective impacts on a wide range of cells and tissues have been reported, recently. Probiotic functions have been shown in various studies including; increasing metabolism, regulating immune system function, improving growth performance, and promoting gastrointestinal (GI) health. These microbes also prevent pathogen infection under in vitro and in vivo conditions, reduce free radical damage, and impede tumor progression.^1^

*Lactobacillus* spp. forms the microbiota of humans and animals, and they colonize the gastrointestinal tract (GIT) and urinary tract. *Lactobacillus* spp. can be found in a wide variety of food products around the world.^2^ The *Lactobacillus* genus consists of many species like as *Lactobacillus plantarum*, *Lactobacillus rhamnosus, Lactobacillus brevis, Lactobacillus reuteri*, and *Lactobacillus rhamnosus* and researchers investigate these species more due to their capability for commercial and industrial health. *L. plantarum* and *L. rhamnosus* are utilized commercially to ferment dairy products, frequently producing foods with enhanced flavor and texture. *L. rhamnosus* is possibly one of the bacterial strains with medical applications that most research has investigated about it recently.^3^, ^4^ Preclinical and animal studies have demonstrated that *Lactobacillus* spp. can help to prevent and treat many GI disorders. These GI disorders include intestinal infections, antibiotic‐associated diarrhea, necrotizing enterocolitis in preterm infants, inflammatory bowel disease (IBD), colorectal cancer, and irritable bowel syndrome.^5^

Inflammation is the response of the immune system to various stimuli such as infections, toxic substances, or irradiation, and it is responsible to eliminate harmful stimuli and initiate the healing process.^6^ As a rule, cellular and molecular reactions limit imminent damage or infection through the occurrence of an acute inflammatory response. Uncontrolled acute inflammation can lead to chronic inflammation.^7^ Many chronic diseases, including cardiovascular and intestinal diseases, diabetes, arthritis, and cancers, are caused by inflammation.^8^ Acute and chronic inflammatory diseases can lead to various health problems and affect patients' quality of life.^9^ One of the most common forms of intestinal inflammation is IBD, which is divided into Crohn's disease (CD) and ulcerative colitis (UC). CD and UC are chronic relapses of the GIT caused by a variety of genetic and environmental factors.^10^ A wide range of pharmacological treatments are available for inflammation‐related diseases, but they are only moderately effective and may be associated with side effects such as drug toxicity.^10^, ^11^ Greater knowledge of inflammatory response pathways and molecular processes is certainly needed for the prevention and treatment of inflammatory diseases.

Autophagy is a catabolic process and it is responsible for the degradation of damaged and dysfunctional organelles. Additionally, the role of autophagy is diverse in different tissues. In the same context, the role of impaired autophagy has been approved in several diseases such as IBD, necrotizing enter colitis, neurodegeneration, and aging.^12^ This pathway includes several stages, including initiation of autophagy, nucleation of autophagosome, extension and maturation of autophagosome, fusion with the lysosome, and breakdown of autophagosome contents.^13^ The autophagy process begins with the creation of a structure called a phagophore that surrounds the products (nucleation stage) and goes towards the formation of autophagosome and then it is formed in the closure stage of autophagosome. This structure breaks down the contents by connecting to the lysosome and using lysosomal hydrolases. This process is complicated and about 40 autophagy‐related proteins (*atg*) are involved in it. In the first step, some genes like *beclin*, *atg14*, *pik3C3* (*vps34*), *pik3R4* (*vps15*), and *atg13* are involved in phagophore formation.^14^ The primary formation of phagophore is essential for the nucleation and elongation phase. There are four ATG proteins named ATG5, ATG 7, ATG 10, and ATG 16 in the autophagosome forming systems, then the ATG5–ATG12 complex is formed and stabilized by ATG16, which leads to the conversion of LC3‐I (cytoplasmic form) to LC3‐II (membrane form) with the assistance of ATG7 and ATG3.^14^, ^15^ In the gut, key functions of autophagy have been reported in innate and adaptive immunity. Deficiency of *ATG16L1* has been shown to affect bacterial clearance in vitro, promoting intracellular bacterial survival, increasing the growth of pathogens, and indicating the secretion of proinflammatory cytokines like interleukin (IL)‐1β and IL‐6.^16^, ^17^

As studies have shown that autophagy plays a role in intestinal homeostasis, defects in autophagy genes have led to a disruption of balance and homeostasis, ultimately causing inflammation in the gut.^18^ We have already demonstrated the phenotypic anti‐inflammatory effects of our probiotic strains in an in vivo study.^19^ Moreover, our recent studies have shown that probiotic can play an effective role to modulate inflammation by affecting on molecular signaling pathways related to inflammation, such as nuclear factor‐κB (NF‐κB), mitogen‐activated protein kinase, and Janus kinases/signal transducer and activator of transcription (JAK‐STAT).^20^, ^21^, ^22^ Currently, our group has decided to determine the effects of *Lactobacillus* spp. on autophagy gene expression to draw a molecular pattern in an in vitro inflammation model.

## 2 MATERIALS AND METHODS

### 2.1 Bacterial cocktail preparation

In the present study, we used a mixture of four different *Lactobacillus* species including *L. plantarum* PR42 and PR365, *L. rhamnosus* PR195, *L. brevis* PR205, and *L. reuteri* PR100 that were isolated from stool and breast milk samples of healthy volunteers as previously reported.^19^, ^23^ These bacteria were grown anaerobically in de man–rogosa–sharpe (MRS) broth at 37°C for 20 h and then were harvested using centrifugation and washed with 0.1 M phosphate buffer (pH 6.9). The bacterial pellet was diluted with antibiotic‐free MRS medium containing 10% fetal bovine serum (FBS), and the optical density 600 nm was adjusted to 0.08 (0.5 McFarland).

### 2.2 The preparation of bacterial strains for inducing inflammation

Enterotoxin‐producing *Escherichia coli* (ETEC) and *Salmonella typhimurium* were cultured in Luria–Bertani broth. Subsequently, the culture medium was heated at 100°C for 10 min, then it was sonicated (10 rounds, 1 min/round). Finally, cell dregs were centrifuged (1700*g*, 15 min, 4°C). In the next step, the human colon adenocarcinoma cell line HT‐29 was provided by the cell bank of the Pasteur Institute of Iran. Roswell Park Memorial Institute (RPMI)‐1640 with 10% FBS and 1% penicillin–streptomycin was prepared to create a nutrient cell medium. For counting the amount of the cells, first 0.25% trypsin‐EDTA separated cells, then they were washed twice with PBS. After centrifuging suspension, the precipitate was diluted with RPMI‐1640, and 2 × 10^5^ cells were seeded per well. HT‐29 cells were exposed to sonicated pathogenic enterotoxin‐producing *E. coli* (SP‐ETEC), and sonicated pathogenic *S. typhi* (SP‐ST).

All protocols were performed under applicable guidelines and regulations, observing the ethical limitations of our previous studies obtained from the committee of the Pasteur Institute of Iran (IR.PII. REC.1398.060).^21^

### 2.3 The investigation of *Lactobacillus* cocktail effects before inflammation

In this step, to investigate the preventive effects of the *Lactobacillus* spp. before inflammation induction, the *Lactobacillus* cocktail was first added to the HT‐29 cell line, and after 6 h, SP‐ETEC and SP‐ST were added to the HT‐29 cell line.

### 2.4 The investigation of *Lactobacillus* cocktail effects concurrently with inflammation

To demonstrate the efficacy of *Lactobacillus* spp. at the beginning of inflammation induction, *Lactobacillus* spp. and SP‐ETEC and SP‐ST were simultaneously added to HT ‐29 cell line.

### 2.5 The investigation of *Lactobacillus* cocktail after inflammation

To designate the effect of the *Lactobacillus* spp. after inflammation induction, SP‐ETEC and SP‐ST were first added to HT‐29 cell line, and then *Lactobacillus* cocktail was added after 6 h to HT‐29 cell line.

Each treatment was accomplished in two separate biological replicates and three separate technical replicates. Each well was washed twice with phosphate buffer to eliminate bacterial particles. These treatments were accomplished twice, and the cell culture flask was preserved at 37°C and 5% CO2 till 48 h later.

### 2.6 Real‐time polymerase chain reaction (PCR) to measure the expression level of genes involved in the autophagy signaling pathway

In the first step, total RNA was extracted according to the manufacturer's protocols (Roche).^24^ The complementary DNA (cDNA) template was synthesized using the cDNA Synthesis Kit (Yekta Tajhiz) according to the manufacturer's instructions. The primer sequences were obtained from the online website PrimerBank (http://pga.mgh.harvard.edu/primerbank) and then purchased from SinaColon Co. (Table 1). All primers were checked using gradient PCR to determine a valid annealing temperature. The messenger RNA quantification of autophagy genes was measured using the ABI Step One Plus detection system (Applied Biosystems) using SYBR Green Master Mix (Amplicon Bio). All tests for each gene were performed in duplicate. The comparative formula in statistics ($RQ=2^{‐∆∆C_{t}}$) was used to obtain the relative gene expression in the comparative CT method.^25^ An internal control gene was required to normalize the data, so glyceraldehyde‐3‐phosphate dehydrogenase was used as a housekeeping gene.^26^, ^27^, ^28^

**Table 1:** List of primers used in this study.

Gene name	Primer forward	Primer reverse
*pik3C3*	CCTGGACATCAACGTGCAG	TGTCTCTTGGTATAGCCCAGAAA
*atg14*	GCAAATCTTCGACGATCCCAT	CACACCCGTCTTTACTTCCTC
*beclin*	CTGGTAGAAGATAAAACCCGGTG	AGGTAGAGCGTGGACTATCCG
*pik3R4*	GGTGGTCACGTTGCTAAGC	CGCAGGTGCCAATCATTCTTAT
*atg7*	CAGTTTGCCCCTTTTAGTAGTGC	CCAGCCGATACTCGTTCAGC
*atg5*	AAAGATGTGCTTCGAGATGTGT	CACTTTGTCAGTTACCAACGTCA
*atg16*	ACCTGCGTGTCAGCAACAT	CAGCTTTGGTCCAGTCAGAAC
*atg3*	ACATGGCAATGGGCTACAGG	CTGTTTGCACCGCTTATAGCA

### 2.7 Cytokine level measurement

To evaluate the expression level of proinflammatory cytokines, including IL‐6 and IL‐1β, the supernatant was prepared after centrifuging at 6000 rpm, the sediment was thrown away, and the supernatant was used for examination with an ELISA kit (Karmanian Pars Gene) according to the manufacturer's instructions.

### 2.8 Statistical comparative analysis

Graph Pad Prism 8 (Graph Pad Software Inc.) was used to study graphs and statistically analyze the data to compare the variables of the different groups. The statistical differences between the different groups, including the control (C), the sonicated pathogen (SP), first the consumption of *Lactobacillus* spp. and secondly the inflammation induction by the sonicated pathogen (LP), first, the induction of inflammation by the sonicated pathogen and second, the consumption of *Lactobacillus* spp. (PL) and the simultaneous administration of *Lactobacillus* spp. and the sonicated pathogen (P + L), were determined using ANOVA. 24 and 48 represent hours of exposing the HT ‐29 cell line to *Lactobacillus* spp. and after that total RNA was extracted from each treatment. *p* < .05 and <.001 were contemplated statistically considerable. Results were expressed as ±standard deviation.

## 3 RESULTS

### 3.1 The effects of *Lactobacillus* spp*.* on the expression of genes involved in phagophore, autophagosome, and autolysosome formation

**Figure 1:**
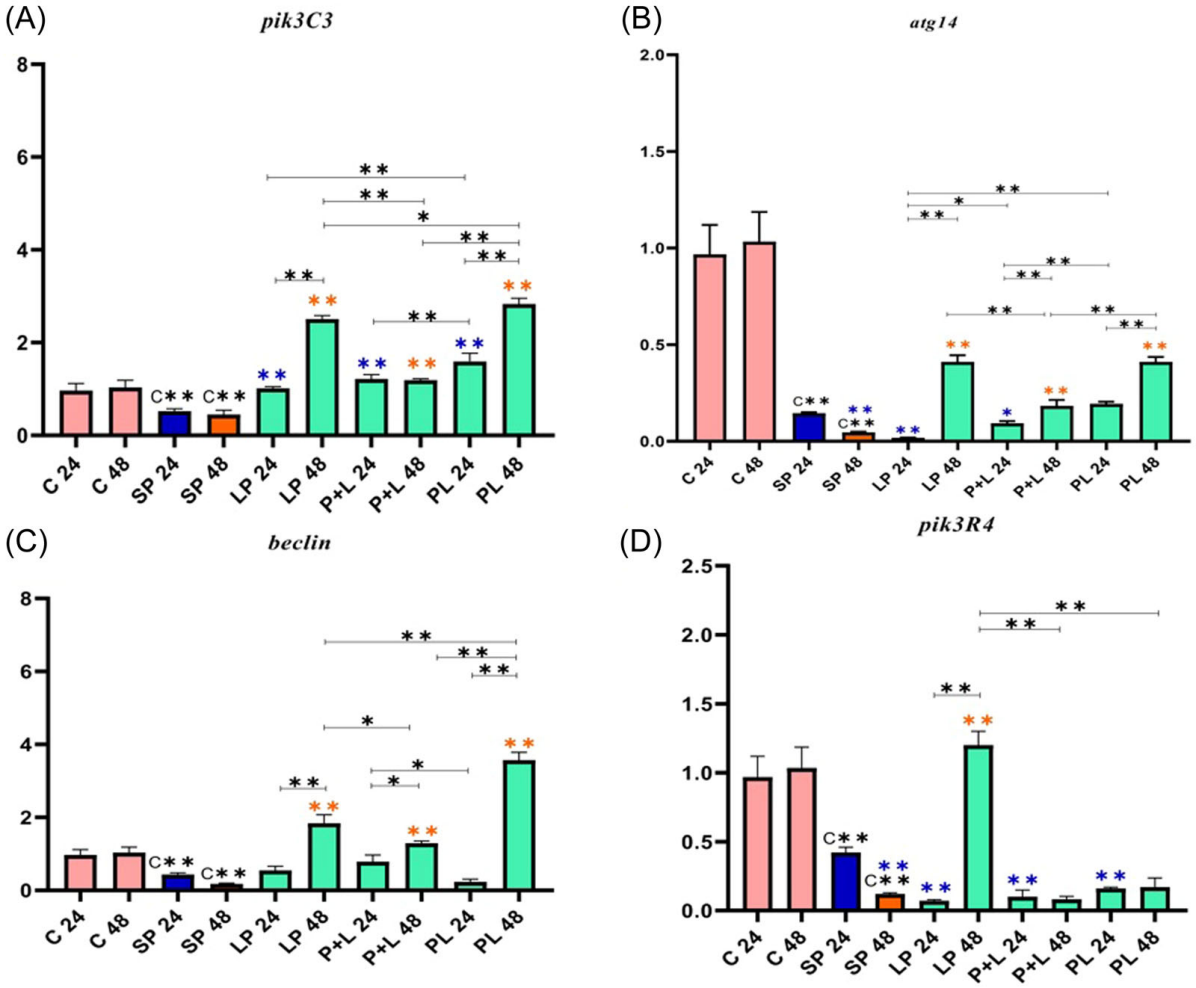
Relative gene expression (mean fold change) of autophagy genes in the different groups of treatments. Data were represented as mean ± SD. Data were considered statistically significant when *p* < .05 (*p < .05, **p < .001). Letters A‐D indicate the graphs of genes involved in phagophore formation (A: *pik3C3*, B: *atg14*, C: *beclin*, D: *pik3R4*). C** shows the relatedness between C24 and C48 with SP 24 and SP 48, the orange color shows the relatedness between SP 24 and other treatments, and the purple color shows the relatedness between SP 48 with other treatments. The relatedness between other treatments is shown with a bracket. SP, sonicated pathogen.

**Figure 2:**
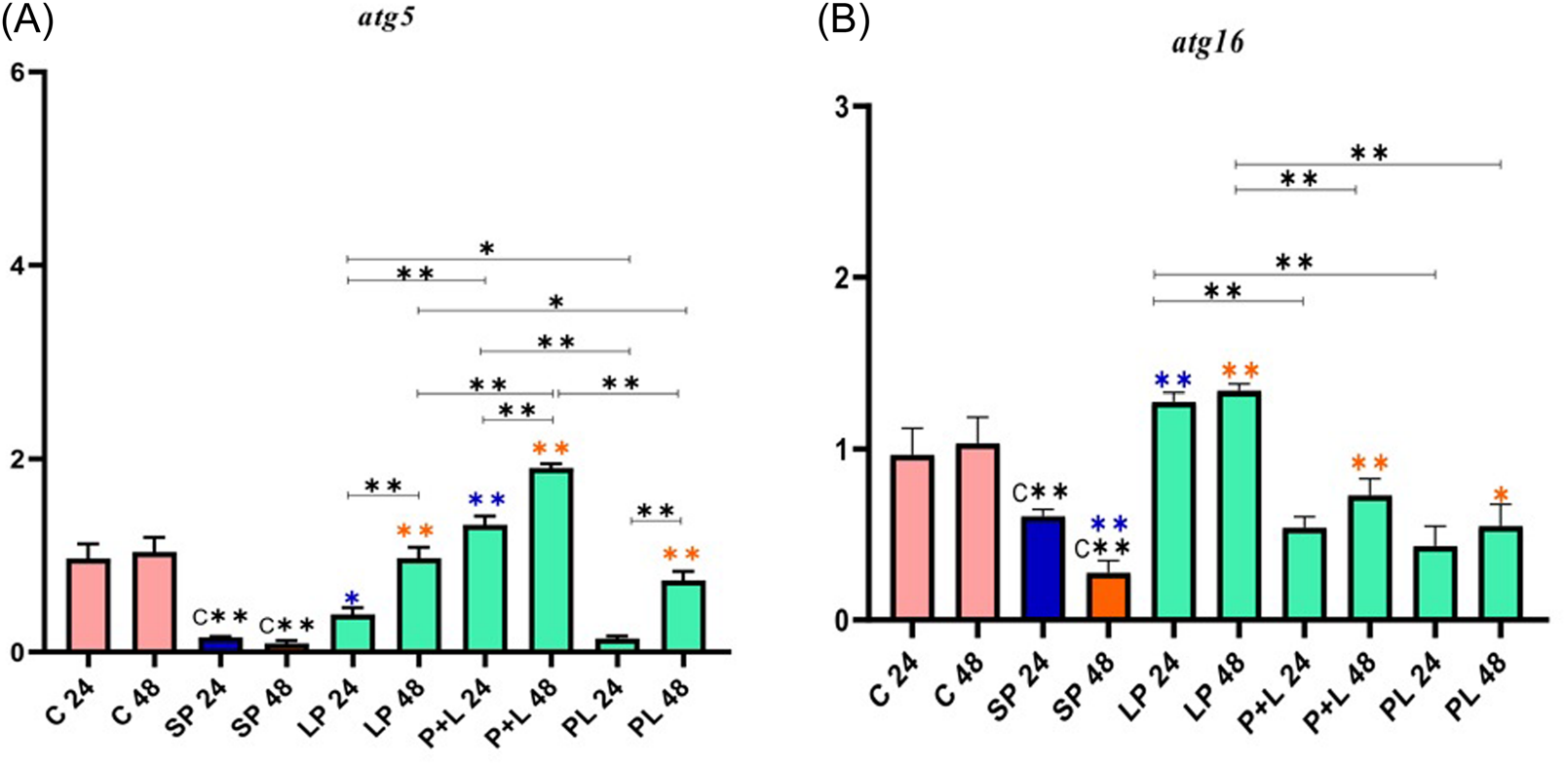
Relative gene expression (mean fold change) of autophagy genes in the different groups of treatments. Data were represented as mean ± SD. Data were considered statistically significant when *p* < .05 (*p < .05, **p < .001). Letters A‐B indicates the graphs of genes involved in autophagosome and autolysosome formation (A: *atg5* and B: *atg16*). C** shows the relatedness between C24 and C48 with SP 24 and SP 48, the orange color shows the relatedness between SP 24 and other treatments, and the purple color shows the relatedness between SP 48 with other treatments. The relatedness between other treatments is shown with a bracket. SP, sonicated pathogen.

**Figure 3:**
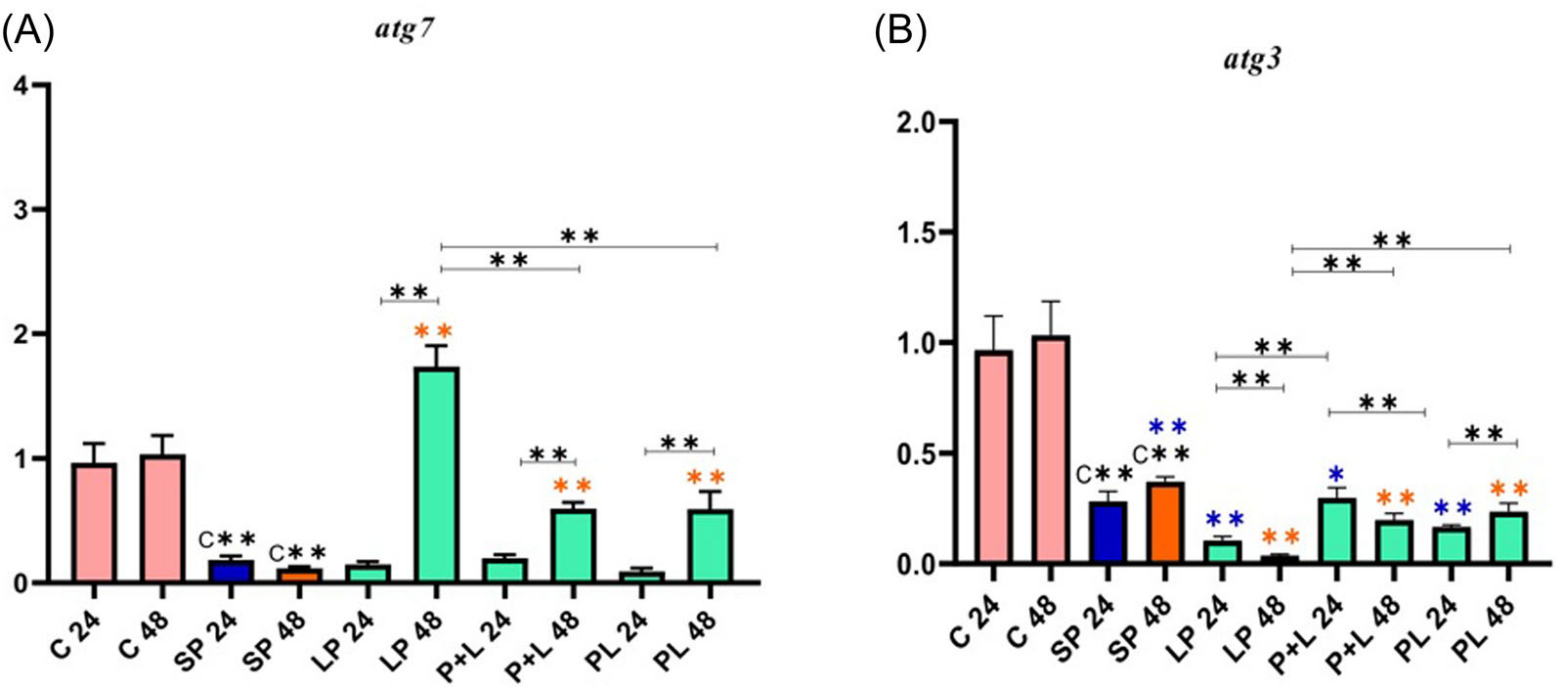
Relative gene expression (mean fold change) of autophagy genes in the different groups of treatments. Data were represented as mean ± SD. Data were considered statistically significant when *p* < .05 (*p < .05, **p < .001). Letters A‐B indicate the graphs of genes involved in autophagosome and autolysosome formation (A: *atg7* and B: *atg3*). C** shows the relatedness between C24 and C48 with SP 24 and SP 48, the orange color shows the relatedness between SP 24 and other treatments, and the purple color shows the relatedness between SP 48 with other treatments. The relatedness between other treatments is shown with a bracket. SP, sonicated pathogen.

Due to the role of genes involved in the autophagy signaling pathway, they were classified into three different categories. First, the expression of genes involved in phagophore formation (e.g., *pik3R4*, *pik3C3*, *atg14*, and *beclin*), second, the expression of genes involved in autophagosome (e.g., *atg5* and *atg16*), and third, the expression of genes involved in autolysosome formation (e.g., *atg7* and *atg3*) was analyzed (see Figures 1, 2, 3).

#### 3.1.1 The effects of *Lactobacillus* spp. on the expression of genes involved in phagophore formation

In the analysis of the *pik3C3*, a decrease in gene expression was observed in SP 24 and SP 48 compared to Control 24 and Control 48 (Figure 1A). An upward trend of gene expression was seen in all treatments and phases. In all 24 h treatments, the highest effect of *Lactobacillus* spp. on *pik3C3* expression was seen in PL 24. In all 48 h treatments, the highest effect of *Lactobacillus* spp. on *pik3C3* expression was seen in PL 48. The comparative analysis showed that there was not a significant increase between P + L 24 and P + L 48, however, a considerable increase was observed in LP 48 in comparison with LP 24, and also in PL 48 compared to PL 24 (*p* < .001). The strongest effect of *Lactobacillus* spp. on *pik3C3* expression belonged to PL 48.

In the comparative analysis of the *atg14*, a decrease in gene expression was observed in SP 24 and SP 48 compared to Control 24 and Control 48 (Figure 1B). A considerable increase was not seen in all 24 h treatments, although, an increasing trend in *atg14* expression was observed in all 48 h treatments. In all 48 h treatments, the highest effect of *Lactobacillus* spp. on *atg14* expression was seen in LP 48 and PL 48. In all phases (pre, simultaneous, and post), gene expression was higher in 48 h treatments compared to 24 h treatments. The strongest effect of *Lactobacillus* spp*.* consumption on *atg14* expression totally occurred before and after inflammation induction (LP 48 and PL 48).

In the statistical analysis of *beclin*, a decrease in gene expression was observed in SP 24 and SP 48 compared to Control 24 and Control 48 (Figure 1C). A significant difference was not observed in all 24 h treatments compared to SP 24. An upward trend of gene expression was seen in all 48 h treatments compared to SP 48. In all 48 h treatments, the highest effect of *Lactobacillus* spp. on *beclin* expression was seen in PL 48. In all phases (pre, simultaneous, and post), a significant increase in *beclin* expression was observed in the 48 h treatments compared to the 24 h treatments (*p* < .001). The strongest effect of *Lactobacillus* spp. on *beclin* expression belonged to the consumption of *Lactobacillus* spp. before and after inflammation induction (LP 48 and PL 48).

In the analysis of the *pik3R4*, a decrease in gene expression was observed in SP 24 and SP 48 compared to Control 24 and Control 48 (Figure 1D). A significant effect of *Lactobacillus* spp. on *pik3R4* expression was not observed in all 24 h treatments. In all 48 h treatments, the highest effect of *Lactobacillus* spp. on *pik3R4* expression was seen in LP 48 (*p* < .001). Totally, the strongest effect of *Lactobacillus* spp*.* on *pik3R4* expression belonged to probiotic consumption before inflammation induction (LP 48).

#### 3.1.2 The effects of *Lactobacillus* spp. on the expression of genes involved in autophagosome formation

In the comparative analysis of the *atg5*, a decrease in gene expression was observed in SP 24 and SP 48 compared to Control 24 and Control 48 (Figure 2A). An increase of *atg5* expression was seen in LP 24 and P + L 24 compared to SP 24 (*p* < .001); however, a significant difference was not observed in PL 24 in comparison with SP 24. In all 24 h treatments, the highest effect of *Lactobacillus* spp. on *atg5* expression was seen in P + L 24. In all 48 h treatments, the highest effect of *Lactobacillus* spp. on *atg5* expression was seen in P + L 48. In all phases (pre, simultaneous, and post), gene expression was higher in 48 h treatments compared to 24 h treatments (*p* < .001). Generally, the strongest effect of *Lactobacillus* spp*.* on *atg5* expression belonged to the probiotic consumption with inflammation induction simultaneously (P + L 48).

In the statistical analysis of *atg16*, a decrease in gene expression was observed in SP 24 and SP 48 compared to Control 24 and Control 48 (Figure 2B). No significant difference was seen in P + L 24 and PL 24 compared to SP 24; however, remarkable increase was observed in LP 24 compared to SP 24. An upward trend of gene expression was seen in all 48 h treatments compared to SP 48 (*p* < .001). In all 24 h treatments, the highest effect of *Lactobacillus* spp. on *atg16* expression was seen in LP 24. In all 48 h treatments, the highest effect of *Lactobacillus* spp. on *atg16* expression was seen in LP 48. In all phases (pre, simultaneous, and post), a significant difference in *atg16* expression was not seen in 48 h treatments compared to 24 h treatments. Finally, the strongest effect of *Lactobacillus* spp*.* on *atg16* expression belonged to the probiotic consumption before inflammation induction (LP 24 and LP 48).

#### 3.1.3 The effects of *Lactobacillus* spp. on the expression of genes involved in autolysosome formation

In the analysis of the *atg7*, a decrease in gene expression was observed in SP 24 and SP 48 compared to Control 24 and Control 48 (Figure 3A). A significant difference was not observed in all 24 h treatments compared to SP 24. An upward trend of gene expression was seen in all 48 h treatments compared to SP 48. In all 48 h treatments, the highest effect of *Lactobacillus* spp. on *atg7* expression was seen in LP 48. In all phases (pre, simultaneous, and post), gene expression was higher in 48 h treatments compared to 24 h treatments (*p* < .001). Totally, the strongest effect of *Lactobacillus* spp. on *atg7* expression belonged to probiotic consumption before inflammation induction (LP 48).

In the analysis of *atg3*, a decrease in gene expression was observed in SP 24 and SP 48 compared to Control 24 and Control 48 (Figure 3B). There was no significant difference in gene expression in different treatments and phases. A decrease in *atg3* expression was observed in the most of treatments. In all 24 h treatments, a decrease in *atg16* expression was seen in LP 24 and PL 24 (*p* < .001) compared to SP 24, however, an increase in *atg3* expression was observed in P + L 24 compared to SP 24 (*p* < .05). Therefore, the strongest effect of *Lactobacillus* spp*.* on *atg3* expression belonged to the probiotic consumption concurrently with inflammation induction (P + L 24).

### 3.2 The results of proinflammatory and inflammatory cytokine production

**Figure 4:**
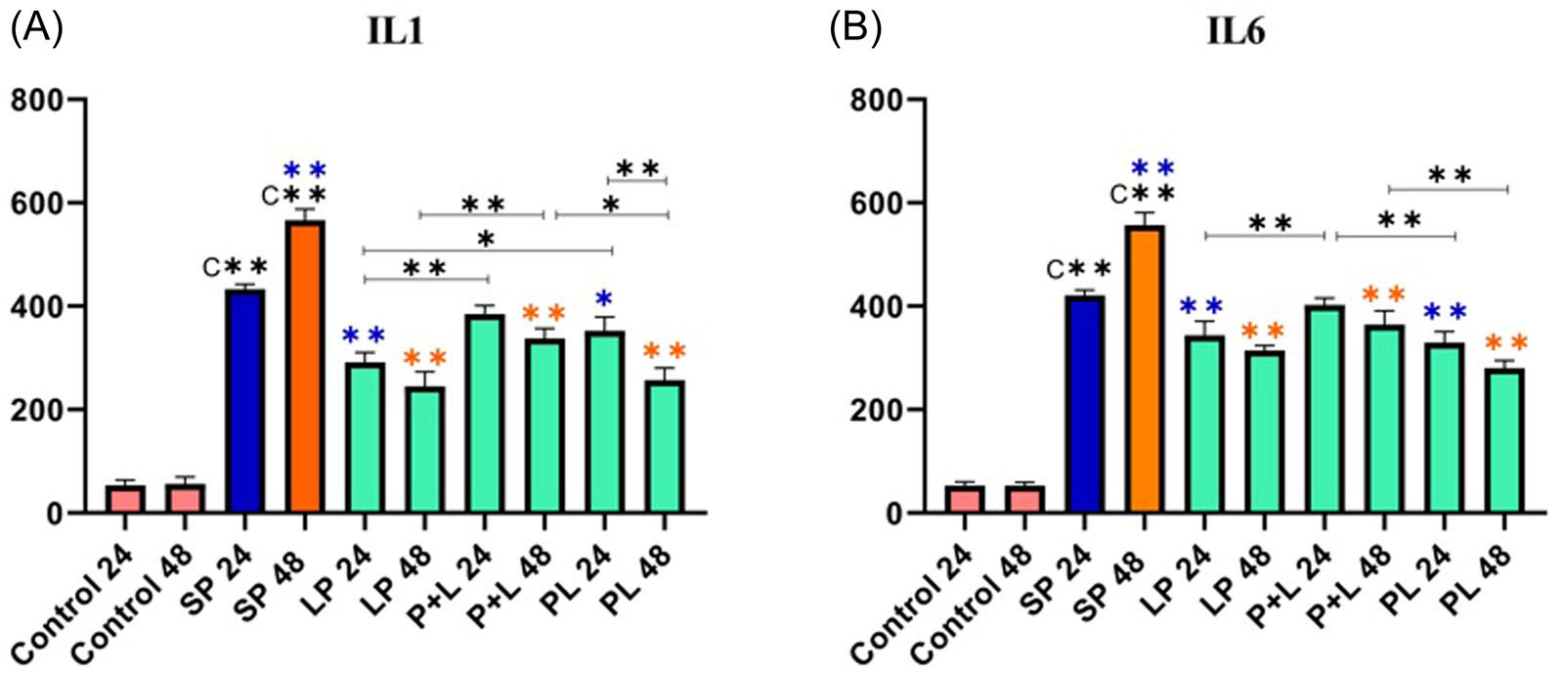
Different levels of concentrations of proinflammatory cytokines (A and B for IL‐1β and IL‐6, respectively). Data were represented as mean ± SD. Data were considered statistically significant when *p* < .05 (*p < .05, **p < .001). C** shows the relatedness between Control 24 and Control 48 with SP 24 and SP 48. The purple color shows the relatedness between SP 24 and other treatments, and the orange color shows the relatedness between SP 48 with other treatments. The relatedness between other treatments is shown with a bracket. IL‐1β, interleukin‐1β; SP, sonicated pathogen.

The result of proinflammatory cytokines production is shown in Figure 4. Cytokine production increased significantly after SP treatments. Although, a significant decrease in cytokine production was observed in treatment with *Lactobacillus* spp. before, after and during inflammation induction. It appears that the consumption of *Lactobacillus* spp. had the greatest effect before and after inflammation in decreasing inflammatory cytokines.

## 4 DISCUSSION

Recently, the terms “gut health” and “gut microbiome” have become popular and they are used mostly in scientific papers, the food industry, and food product advertisements.^29^ Probiotics are crucial in treating infectious diseases, GI issues, and inflammation. The gut microbiota has diverse roles in immunity, endocrine function, and maintaining organ health, highlighting the importance of the gut‐organ axis.^30^ One of the important mechanism that is related to inflammation is autophagy signaling pathways that are vital in maintaining tissue homeostasis in different organs. Mutation or deletion in autophagy genes, such as *atg7*, can lead to Paneth cell dysfunction, elevated levels of IL‐1β and TNF‐α, and activation of T helper 17 by dendritic cells.^31^, ^32^ The loss of *atg16* leads to increased IL‐18 and IL‐1β production, enhanced TNF‐α‐induced apoptosis, and disrupts intestinal homeostasis, contributing to the pathogenesis of CD.^33^, ^34^, ^35^ Based on the advantageous impacts of *Lactobacillus* on inflammation reduction, we intended to investigate the role of *Lactobacillus* spp. on the autophagy signaling pathway to provide a molecular scheme for the prevention and treatment of inflammation‐related diseases.

In this study, we investigated the role of *Lactobacillus* spp*.* in autophagy activation by using molecular techniques focused on the expression of different autophagy genes. The present study shows that probiotics can have an effective role in all stages of autophagy. Several studies have shown that probiotic administration can influence autophagy by enhancing the expression of autophagy‐related genes like *atg5*, *atg7*, *atg5*–*atg12*–*atg16* complex, and *beclin*. This modulation of autophagy by probiotics has been linked to various beneficial effects such as promoting survival, enhancing growth performance, maintaining gut homeostasis, protecting intestinal mucus layer function, exerting anti‐inflammatory activity in intestinal tissues and cell lines, and inducing cell death in tumor cells.^36^

In the phagophore stage and autophagosome stage, *Lactobacillus* spp. can be able to prevent and reduce inflammation by increasing the autophagy gene expression. In the autolysosome stage, the preventive role of *Lactobacillus* spp. can be deduced by the analysis of *atg7*. A significant inference was not observed from *atg3*, but in the postphase, there was an increasing trend in PL 48 compared to PL 24. Hence, in *atg3*, we hope that the therapeutic role of *Lactobacillus* spp. will be determined by investigating treatments after 72 and 96 h. In *atg3,* more period of time should be considered for the investigation of *Lactobacillus* spp. effect on gene expression. Totally, the highest effect of *Lactobacillus* spp. on gene expression was seen before inflammation induction, so probiotics can be effective for the prevention of inflammation. Lu et al. showed the anti‐inflammatory and autophagy‐inducing properties of lactic acid bacteria in combating infection and inflammation, especially with affecting *atg16* expression.^37^ Bajić et al. showed that *Lactobacillus brevis* BGZLS10‐17 exhibits immune‐regulatory effects through influencing *atg5*‐dependent autophagy in vitro.^38^

According to cytokines measurement, the addition of sonicated pathogens could remarkably increase the expression level of proinflammatory and inflammatory cytokines. This study showed that pathogenic components can cause inflammation. *Lactobacillus* spp. was added to all treatments and a significant reduction of inflammation was observed by IL‐6 and IL‐1β measurement in all phases. In addition, the strongest effect of *Lactobacillus* spp. on the reduction of IL‐6 and IL‐1β was seen when probiotics were added before and after inflammation of the cell cultures. The role of autophagy has been evaluated by Wang et al. and they have shown that activation of autophagy pathways increased the anti‐inflammatory cytokine IL‐10, so *Lactobacillus* spp. has an important effect on hemostasis by inducing anti‐inflammatory cytokine can regulate immune system in the mucosal membrane.^39^ Whereas, inhibition of autophagy led to a strong increase in IL‐1β secretion. *L. reuteri* 100‐23, *L. gasseri* IPL A6.33, and *L. rhamnosus* IPL A2.21 increased the production of IL‐1β, followed by *atg16L1* deficiency that was first reported by Saitoh et al.^34^, ^40^ The reduction of *atg16* expression activates the inflammatory pathways. As a result, the expression of *IL‐1β* and *IL‐18* genes will increase when *atg16* expression is decreased.^40^

Another interesting outcome of this study was the significant increase in gene expression in 48 h treatments compared to 24 h treatments, so the next studies should try to consider the role of probiotics in long‐term consumption in IBD models. Another important point to mention is that in our recent study by Ghanavati et al., the β‐catenin protein level was assessed. The study revealed that the use of our native *Lactobacillus* strains resulted in a reduction of this protein in the HT‐29 cell line.^41^ This finding further supports our current research, which focuses on activating autophagy signaling genes by increasing their expression levels. Because, according to Su et al., there is a cross‐talk between the Wnt/β‐catenin and autophagy signaling pathways and this interaction leads to the induction of autophagy through the silencing of β‐catenin.^42^ Hence, one could infer that our native *Lactobacillus* strains may influence different networks and produce anti‐inflammatory outcomes. There are numerous genes that involved in the autophagy signaling pathway; therefore, the other important genes including *atg4*, *atg10*, and *atg12* can be investigated in future studies. New approaches for treating inflammatory‐related diseases should be evaluated including exploring various probiotics strains, other signaling and immunological pathways. A recent study investigated the impact of *Lactobacillus* spp. on inflammation and autophagy gene expression, providing insights into the potential use of probiotics for preventing and treating IBD. Further research is required to fully assess this potential treatment option.

Based on the advantageous impacts of probiotics, it can be concluded that probiotics have multilateral role in maintaining gut hemostasis and affect various signaling pathways, including JAK/STAT, NF‐κB, mitogen‐activated protein kinase, and cell junction through upregulation of *cx26* and *cx43* expression.^43^, ^44^ Therefore, we hope that provide a broader molecular scheme for the prevention and treatment of inflammation‐related diseases by extending our research in the future to evaluate multilateral role of *Lactobacillus* spp. on different signaling pathways leading to inflammation. Thus, the anti‐inflammatory properties of probiotics could be effective not only in inflammation modulation in intestinal tissue but also in other organs especially the brain through the gut–brain–axis. In this research, it has been shown that the consumption of *Lactobacillus* spp. before, simultaneously, and after inflammation induction, ultimately leading to the activation of the autophagy signaling pathway.

The research conducted has primarily concentrated on the molecular characteristics of the anti‐inflammatory and autophagy‐inducing properties of native probiotics in an in vitro model of IBD, as mentioned earlier. Limitations do exist within the current study. As previously mentioned, various proteins such as LC3‐I, LC3‐II, and ATG16 are involved in the autophagosome formation and elongation phase of the autophagy signaling pathway. Thus, assessing the phosphorylation of these proteins may offer deeper insights into the activation of this particular pathway. Due to the emphasis of the current study on molecular analysis, the investigation into the impact of native probiotic strains on the protein levels of autophagy was not conducted. Future research endeavors should prioritize protein level assessments, including western blot analysis, in addition to molecular experiments, to enhance our understanding of the beneficial attributes of native *Lactobacillus* spp. and *Bifidobacterium* spp. in combating inflammation through autophagy stimulation.

## 5 CONCLUSION

Finally, understanding the molecular effects of *Lactobacillus* spp. on the autophagy signaling pathway could shed light on anti‐inflammatory and immune‐modulatory properties of probiotics that can be effective in the inflammation reduction. It is critical to investigate the anti‐inflammatory properties of probiotics on other signaling pathways that lead to inflammation to present a comprehensive concept for administering probiotics as a supplementary agent in inflammatory disorders. In this research, *Lactobacillus* spp. consumption before, simultaneously, and after inflammation showed beneficial effects on inflammation reduction. Our results illustrated that these probiotic strains could trigger an autophagy signaling pathway to target the inflammation, but more molecular investigation is needed to demonstrate our isolated probiotic potency in the prevention of the inflammation pathogenesis and alleviating inflammation severity in inflammatory diseases.

## AUTHOR CONTRIBUTIONS

*Performed the experiments*: Fatemeh Haririzadeh Jouriani, Mahnaz Torfeh, and Mahdi Torkamaneh. *Data analysis*: Amin Sepehr, Fatemeh Haririzadeh Jouriani, and Mahdi Torkamaneh. *Writing of the manuscript*: Fatemeh Haririzadeh Jouriani. *Revised manuscript*: Shadi Aghamohammad and Mahdi Rohani. *Conceived and designed the experiments*: Shadi Aghamohammad and Mahdi Rohani.

## CONFLICT OF INTEREST STATEMENT

The authors declare no conflict of interest.

## ETHICS STATEMENT

The experimental protocols were established following the Declaration of Helsinki and approved by the Ethics Committee of the Pasteur Institute of Iran (IR.PII.REC.1398.060). All methods were carried out in accordance with relevant guidelines and regulations.

## Data Availability

The datasets generated during and/or analyzed during the current study are available from the corresponding author upon reasonable request.
